# Investigating the Mechanism of Cadmium-Tolerant Bacterium *Cellulosimicrobium* and Ryegrass Combined Remediation of Cadmium-Contaminated Soil

**DOI:** 10.3390/plants13121657

**Published:** 2024-06-15

**Authors:** Jiaqi Li, Xiaoyang Xu, Lanping Song, Meng Na, Shangqi Xu, Jie Zhang, Yongjie Huang, Xiaoping Li, Xianqing Zheng, Jihai Zhou

**Affiliations:** 1 School of Ecology and Environment, Anhui Normal University, Wuhu 241002, China; krieben@163.com (J.L.); xxy18856023887@163.com (X.X.); songlanping98@163.com (L.S.); nameng1991@ahnu.edu.cn (M.N.); shangqixu@ahnu.edu.cn (S.X.); zhjie1211@sina.com (J.Z.); yongjiehuang0108@163.com (Y.H.); 2 Collaborative Innovation Center of Southern Modern Forestry, Nanjing Forestry University, Nanjing 210037, China; xpli@njfu.edu.cn; 3 Institute of Eco-Environment and Plant Protection, Shanghai Academy of Agricultural Sciences, Shanghai 201403, China

**Keywords:** Cd pollution, biological co-remediation, antioxidant enzyme system, none-enzymatic antioxidant system, microorganisms

## Abstract

Cadmium (Cd) pollution has been rapidly increasing due to the global rise in industries. Cd not only harms the ecological environment but also endangers human health through the food chain and drinking water. Therefore, the remediation of Cd-polluted soil is an imminent issue. In this work, ryegrass and a strain of Cd-tolerant bacterium were used to investigate the impact of inoculated bacteria on the physiology and biochemistry of ryegrass and the Cd enrichment of ryegrass in soil contaminated with different concentrations of Cd (4 and 20 mg/kg). The results showed that chlorophyll content increased by 24.7% and 41.0%, while peroxidase activity decreased by 56.7% and 3.9%. In addition, ascorbic acid content increased by 16.7% and 6.3%, whereas glutathione content decreased by 54.2% and 6.9%. The total Cd concentration in ryegrass increased by 21.5% and 10.3%, and the soil’s residual Cd decreased by 86.0% and 44.1%. Thus, the inoculation of Cd-tolerant bacteria can improve the antioxidant stress ability of ryegrass in Cd-contaminated soil and change the soil’s Cd form. As a result, the Cd enrichment in under-ground and above-ground parts of ryegrass, as well as the biomass of ryegrass, is increased, and the ability of ryegrass to remediate Cd-contaminated soil is significantly improved.

## 1. Introduction

Heavy metal pollution has been turning into a serious environmental concern across the world. About 19.4% of China’s arable land (2.6 × 10^7^ hm^2^ area) is polluted by heavy metals [1]. According to the Report of the Ministry of Ecology and Environment on the Status of Ecological Environment in China in 2021, the main pollutants that affect the soil’s environmental quality in agricultural land are heavy metals, among which cadmium is the primary pollutant [2]. Various heavy metals, such as cadmium, lead, chromium, and mercury, are toxic to most organisms at higher concentrations [3]. According to the literature, exposure to heavy metals has been linked to a variety of human diseases [4,5,6], such as emphysema, kidney failure, osteoporosis, and cancer [7,8,9]. Therefore, heavy metal remediation is emerging as a serious problem that needs to be effectively addressed.

Compared with physical remediation and chemical remediation, soil biological remediation has the comparative advantages of optimizing and improving the environment at a low cost. For this reason, it has become the main remediation method for cadmium-contaminated soil [10]. Phytoremediation refers to planting a plant on soil polluted by heavy metals to absorb heavy metals in the soil and achieve the purpose of soil remediation [11]. Ryegrass is often used in the remediation of heavy metal-contaminated soil, as it is an excellent tolerant herb with a fast growth rate, high yield, long growing season, and wide adaptability [12,13]. It has been demonstrated that ryegrass can effectively accumulate heavy metals, such as Cd [14,15]. However, the scope of simple phytoremediation is limited, and excessive absorption of heavy metals will have a large number of negative effects on plants and even lead to plant death [16,17]. On the contrary, the inoculation of microorganisms can promote plant growth and improve the efficiency of bioremediation. During the combined plant–microbial remediation, two aspects mainly govern the mechanism of microbial action. Microorganisms can regulate the absorption and accumulation of heavy metals by plants by changing the form of the heavy metals and the growth of plant roots [18]. Thus, the extraction capacity of heavy metals by plants can be improved and the removal of heavy metals from soils can be enhanced. On the other hand, the regionalization, chelation, and fixation of heavy metals in microbial cells can reduce the migration ability of the heavy metals [19], which is conducive to the fixation of heavy metals in plants. At the same time, the growth of plant roots could secrete protein, sugar, and other organic matter as a source of nutrients and energy for microorganisms and, at the same time, significantly improve the activity and adaptability of microorganisms [20,21]. Plants and microorganisms form symbionts for mutual benefit, which strengthens their respective roles in the remediation of heavy metal-contaminated soil and improves the removal efficiency. The presence of Cd in soil can cause damage to plants, such as damage to the antioxidant system, changes in membrane lipid catalase, enzyme activity, etc. [22]. Therefore, the physiological and biochemical characteristics of plants can be used as indicators to evaluate plant growth and repair efficiency. Soil microorganisms can regulate the decomposition of both soil organic matter and harmful compounds and participate in the biochemical cycle and the formation of the soil structure. In parallel, they are sensitive to external interference. Therefore, soil enzyme activity is considered a good index to detect soil quality [23].

To explore the impact of plant–microbial remediation, in this work, the ryegrass and Cd-tolerant bacteria were selected as remediation materials for cadmium-contaminated soil. Ryegrass and Cd-tolerant bacteria were used to study the changes in the physiological and biochemical characteristics of ryegrass, including the biomass, chlorophyll content, and antioxidant enzymes, as well as the changes in soil microbial activity and cadmium enrichment in ryegrass in an indoor pot experiment. The physiological response of ryegrass and the changes in the soil quality due to Cd pollution were thoroughly investigated to provide theoretical guidance and a practical basis for the remediation of Cd-contaminated soil.

## 2. Results

### 2.1. Changes in Growth, Physiology, and Biochemistry of Ryegrass

#### 2.1.1. Changes in Biomass and Chlorophyll Content of Ryegrass

**Figure 1:**
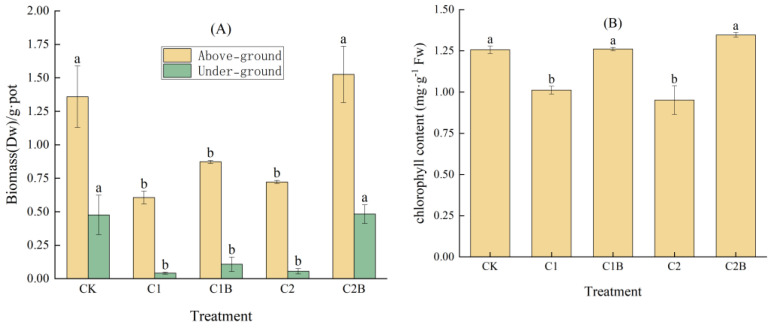
Changes in biomass (**A**) and chlorophyll content (**B**) of *Lolium perenne*.

The above-ground and under-ground biomass of C1 and C2 were 55.4% and 46.8%, 91.3% and 88.3% lower than CK, respectively. Under the treatment of 4 mg/kg Cd, the biomass of above-ground and under-ground in C1B increased by 44.1% and 159.6%, respectively, compared to that of C1. For the concentration of 20 mg/kg Cd, the above-ground and under-ground biomass of C2B significantly increased by 111.1% and 765.2%, respectively, compared to C2 (Figure 1A). Compared to CK, the chlorophyll content of C1 and C2 decreased by 19.5% and 24.3%, respectively. In general, the inoculation of Cd-tolerant bacteria could increase the chlorophyll content of ryegrass. Under the treatment of 4 mg/kg Cd, the chlorophyll content of C1B significantly increased by 24.7% compared to that of C1. Under the treatment of 20 mg/kg Cd, the chlorophyll content of C2B greatly increased by 41.0% compared to that of C2 (Figure 1B).

CK is the control treatment. C1 is the treatment with 4 mg/kg Cd pollution. C2 is the treatment with 20 mg/kg Cd pollution. C1B is the treatment with 4 mg/kg Cd pollution and Cd-tolerant bacteria. C2B is the treatment with 20 mg/kg Cd pollution and Cd-tolerant bacteria. The different letter indicates significant differences between treatments (*p* < 0.05).

#### 2.1.2. Changes in SOD and POD Activities of Ryegrass

**Figure 2:**
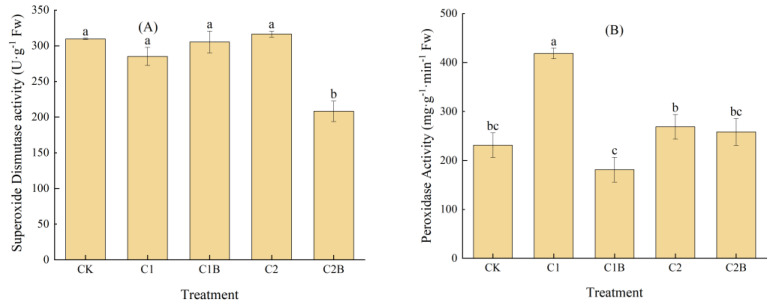
Changes in SOD (**A**) and POD (**B**) activity of ryegrass.

The SOD activity of C1 ryegrass decreased by 7.9% compared to CK, while C2 increased by 2.1%. Under the application of a 4 mg/kg Cd treatment, C1B increased by 7.1% compared to C1. For the concentration of 20 mg/kg Cd, the SOD activity of C2B significantly declined (34.2%) compared to that of C2 (Figure 2A). The POD activity of C1 significantly increased by 81.1% compared to CK, while that of C2 increased by 16.2%. The POD activity of ryegrass declined due to the inoculation of Cd-tolerant bacteria. For a 4 mg/kg Cd concentration, C1B significantly decreased by 56.7% compared to C1, whereas for a 20 mg/kg Cd concentration, C2B reduced by about 3.9% compared to C2 (Figure 2B).

#### 2.1.3. Changes in ASA and GSH Contents of Ryegrass

**Figure 3:**
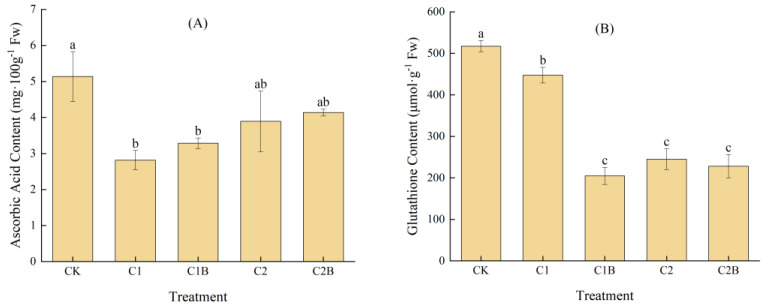
Changes in ASA (**A**) and GSH (**B**) content of ryegrass.

Under Cd stress, the ASA content significantly decreased by 45.2% in C1 and 24.2% in C2 compared to CK. The inoculation with Cd-tolerant bacteria could increase the ASA content in ryegrass. At a 4 mg/kg Cd concentration, the ASA content in C1B increased by about 16.7% compared to C1 with no significant difference. At a 20 mg/kg Cd concentration, C2B increased by 6.3% compared to C2 with no significant difference (Figure 3A). The Cd stress can also reduce the GSH content. More specifically, the GSH contents of C1 and C2 were significantly reduced by 13.5% and 52.6% compared to CK. The GSH content in ryegrass decreased by inoculation with the Cd-tolerant bacteria. Under a 4 mg/kg Cd treatment, the GSH content in C1B significantly decreased by 54.2% compared to C1. At 20 mg/kg Cd concentration, C2B decreased by 6.9% compared to C2 (Figure 3B).

### 2.2. Changes in Soil Basal Respiration and Enzyme Activity

**Figure 4:**
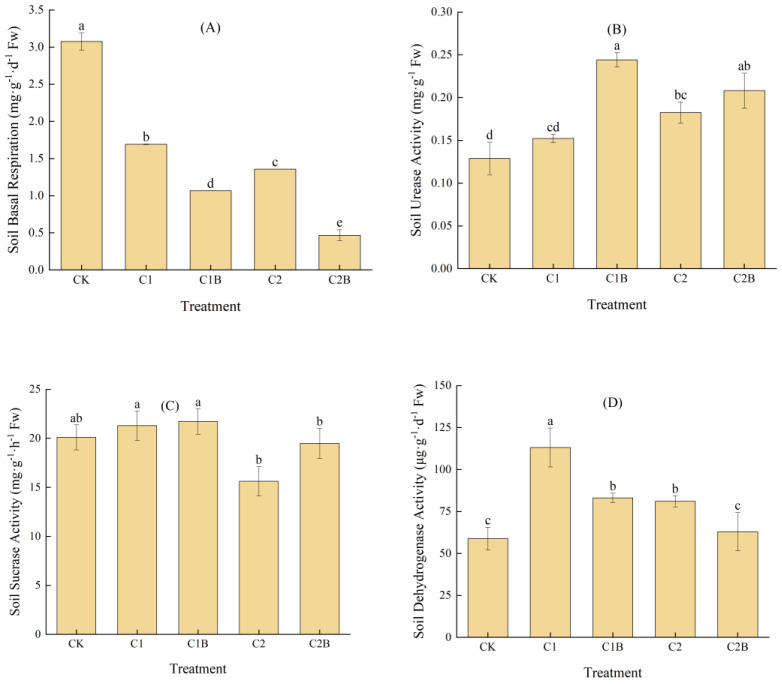
Changes in soil basal respiration (**A**), urease activities (**B**), sucrase activity (**C**), and dehydrogenase activity (**D**).

The Cd stress can reduce the intensity of soil basal respiration. Particularly, the soil basal respiration of C1 and C2 significantly decreased by 45.0% and 55.9% compared to CK. The inoculation of Cd-tolerant bacteria could reduce the soil basal respiration intensity. Under 4 mg/kg Cd treatment, the soil basal respiration intensity of C1B significantly reduced by about 36.9% compared to C1. For the 20 mg/kg Cd concentration, C2B significantly reduced by 65.6% compared to C2 (Figure 4A).

The Cd stress can also lead to an increased soil urease activity. In more detail, the soil urease activity of C1 increased by 18.1% compared to CK, but no significant difference was detected. The soil urease activity of C2 significantly increased by 41.7% compared to CK. The inoculation of Cd-tolerant bacteria can improve soil urease activity. At a 4 mg/kg Cd concentration, the soil urease activity of C1B considerably increased by 60.4% compared to C1. At a 20 mg/kg Cd concentration, the soil urease activity of C2B increased by 14.0% compared to C2 while having no significant difference (Figure 4B).

Soil sucrase activity of C1 increased by 5.9% compared to CK and C2 decreased by 22.3%. The inoculation of Cd-tolerant bacteria could improve soil sucrase activity. At a 4 mg/kg Cd concentration, the soil sucrase activity of C1B was 2.0% higher than C1 while having no significant difference. At a 20 mg/kg Cd treatment, the soil sucrase activity of C2B was 24.8% higher than C2 with no significant difference (Figure 4C).

The Cd stress can increase soil dehydrogenase activity. The soil dehydrogenase activity of C1 and C2 significantly increased by 92.2% and 37.6% compared to CK. The inoculation of Cd-tolerant bacteria decreased the soil dehydrogenase activity. At a 4 mg/kg Cd concentration, the soil dehydrogenase activity of C1B significantly decreased by 26.6% compared to C1. At a 20 mg/kg Cd concentration, the soil dehydrogenase activity of C2B significantly reduced by 22.3% compared to C2 (Figure 4D).

### 2.3. Effects of Cd-Tolerant Bacteria on Soil Cd Forms and Cd Accumulation in Ryegrass

#### 2.3.1. The Content and Changes in Soil Cd Forms

The inoculation of Cd-tolerant bacteria could change the soil’s Cd forms (Table 1). Under a 4 mg/kg Cd treatment, the proportion of exchangeable Cd in C1B increased by 4.19%, the proportion of potentially bioavailable Cd increased by 15.3%, and the proportion of residual Cd decreased by 86.0% compared to C1. The inoculation of Cd-tolerant bacteria could increase the contents of exchangeable and potentially bioavailable Cd in the soil to some extent and reduce the residual Cd content. Under the treatment of 20 mg/kg Cd, the proportion of exchangeable Cd in C2B decreased by about 4.56% and the proportion of residual Cd decreased by about 44.1% compared to C2. In striking contrast, the proportion of potentially bioavailable Cd increased by 26.5%.

#### 2.3.2. Changes in the Cd Content of Ryegrass

**Figure 5:**
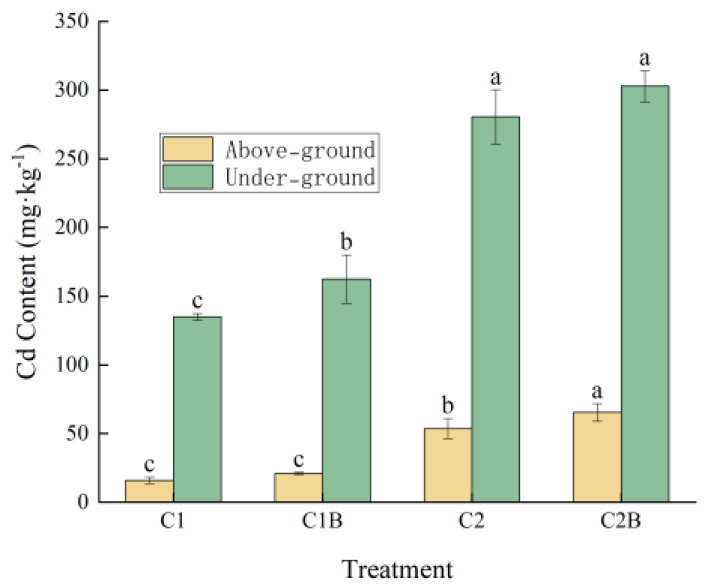
Cd Enrichment in Ryegrass.

The inoculation of Cd-tolerant bacteria could increase the concentration of Cd accumulation in ryegrass, which leads to a reduced content of residual Cd in the soil. More specifically, under the application of a 4 mg/kg Cd concentration, the Cd content above-ground in C1B was 31.9% higher than that in C1 and 20.3% higher than that in C1 under-ground. Under the application of a 20 mg/kg Cd concentration, the Cd content of C2B in the above-ground significantly increased by 22.2% compared to C2 and the under-ground part increased by 8.0% (Figure 5).

### 2.4. Correlation of Cd Accumulation in Ryegrass with Microorganisms, Physiology of Ryegrass

**Figure 6:**
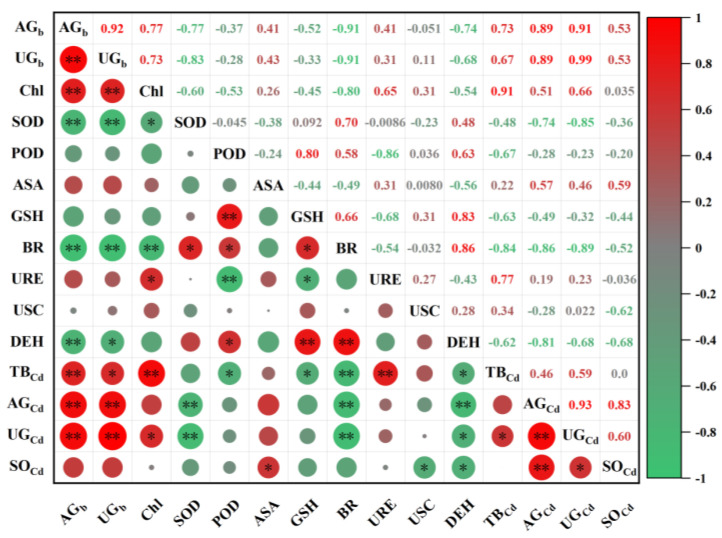
Correlations between the accumulation amount of Cd, physiological characteristics of ryegrass, and soil enzyme activities. AG_b_—above-ground biomass, UG_b_—under-ground biomass, Chl—chlorophyll content, SOD—superoxide dismutase, POD—peroxidase, ASA—ascorbic acid, GSH—glutathione, BR—soil basal respiration, URE—soil urease, USC—soil sucrase, DEH—soil dehydrogenase, TB_Cd_—tolerant bacteria, AG_Cd_—the accumulation amount of Cd in above-ground, UG_Cd_—the accumulation amount of Cd in under-ground, SO_Cd_—soil Cd content. * indicates *p* ≤ 0.05, ** indicates *p* ≤ 0.01.

The inoculation of Cd-tolerant bacteria remarkably increased the total amount of Cd enriched in the under-ground of ryegrass and improved the enrichment in the above-ground (Figure 6). The concentration of Cd was found to be positively correlated with the biomass of ryegrass. The content of chlorophyll was found to be positively correlated with above-ground and under-ground biomass. On the contrary, the SOD activity was negatively correlated with the concentration of cadmium. ASA was positively correlated with Cd enrichment, while GSH was inversely correlated. Moreover, the soil basal respiration and soil dehydrogenase activity were negatively correlated with Cd enrichment.

## 3. Discussion

### 3.1. Effects of Cd-Tolerant Bacteria Inoculation on the Physiology and Biochemistry of Ryegrass

Biomass is a comprehensive index to measure the strength of plant tolerance. The increase in plant biomass in a polluted environment can be a direct indicator that plants have a stronger tolerance to pollution [24]. It has been reported in the literature that a certain degree of heavy metal pollution could directly lead to a decline in biomass. The existence of a higher concentration of heavy metals in the soil leads to a bigger toxicity to plants and greatly affects the plant biomass. The total biomass of organisms under the presence of the Cd stress could be significantly decreased [25]. Zhang et al. [26] found that inoculation with different PGPRs could promote the growth and photosynthesis of *P. praeruptorum* to different degrees, thereby improving its tolerance to the environment. Hu et al. [27] pointed out that arbustic mycorrhizal fungi could improve Alfred stonecrop (*Sedum alfredii* Hance) and fast-growing perennial ryegrass (*Lolium perenne* L.) under Cd-contaminated conditions biomass. In this work, the biomass of ryegrass was increased by the inoculation of Cd-tolerant bacteria. The origins of this effect could be ascribed to the fact that the inoculation of Cd-tolerant bacteria alleviated the toxic effect of Cd on ryegrass. As a result, the chlorophyll content increased, and the photosynthesis improved.

Chlorophyll is considered the basis of plant photosynthesis, and its content is closely related to plant growth and development. It has been demonstrated in the literature that the existence of a Cd stress has a negative impact on chlorophyll content, which is gradually decreased with the increase in the Cd concentration in the soil [28,29]. In this work, the chlorophyll content under Cd stress decreased compared to the case where there was no Cd stress. This effect indicated that Cd had an adverse impact on photosynthesis because the inflow of Cd^2+^ to plant cells could replace the cations related to chlorophyll synthesis in chloroplasts (ex: Fe^2+^, Zn^2+^, and Mg^2+^). It could also bind to proteins containing SH in chloroplasts, inhibit the synthesis of chlorophyll precursors, destroy chloroplast structure, and ultimately reduce chlorophyll content [30,31]. El-Saadony et al. [32] found that inoculation with PGPR increased the chlorophyll content of pepper (*Capsicum annuum* L.) in Cd-contaminated soil. In this work, the inoculation of Cd-tolerant bacteria increased the chlorophyll content of ryegrass leaves, which could be attributed to the fact that Cd-tolerant bacteria enhanced the absorption of K and Fe in ryegrass and promoted the synthesis of chlorophyll. This assumption is also consistent with the results of previously reported works [33].

The antioxidant activity of various enzymes, such as SOD, plays a crucial role in protecting plants from oxidative stress-induced damage caused by abiotic factors, such as Cd stress [34]. It has been proven that the relative increase or decrease in antioxidant enzyme activity may vary based on species and the level of heavy metal pollution in the soil [35,36,37]. SOD is considered an important part of the plant’s antioxidant system. To this end, it has been shown that the SOD activity of tobacco leaves decreases under the existence of a low Cd concentration [38], and a high Cd concentration can inhibit the enzyme activity or protein expression of SOD [39]. In this experiment, the SOD activity was not significantly increased under stress, and the activity was relatively stable. This effect could be attributed to the fact that the Cd concentration in the soil environment did not reach the threshold value of stress on ryegrass, and the SOD activity did not significantly change. This outcome is also in direct line with the literature [40]. The inoculation of Cd-tolerant bacteria under high Cd concentrations can reduce the SOD activity. The latter stems from the inoculation of Cd-tolerant bacteria under Cd stress, which can further alleviate the toxic effect of Cd on ryegrass and reduce the accumulation of reactive oxygen species in ryegrass, resulting in a decline in the SOD activity. POD is a highly active enzyme that can decompose H_2_O_2_ into O_2_ and H_2_O in plants, which is related to photosynthesis, respiration, and the auxin oxidation reaction. It is a common reference index in plant aging and stress. It has been reported that, under the existence of Cd stress, the POD content of Lemna minor could be increased and then decreased with the increase in the Cd concentration [41]. The increase in the POD content at low Cd concentrations further protected plants from peroxidation damage. At high Cd concentrations, the POD activity decreased, possibly because the high Cd content inhibited the enzyme synthesis or subunit assembly of enzymes [42]. The reduction in the POD activity after inoculation with Cd-tolerant bacteria could be ascribed to the decrease in free radical accumulation in plants after the inoculation with Cd-tolerant bacteria. As a result, the degree of oxidative damage to plants was reduced, and the POD activity was reduced to regulate the adaptive ability of plants.

The ascorbate–glutathione cycle is also considered an important component of the antioxidant system in plants. Ascorbic acid reduces H_2_O_2_ to H_2_O via the catalysis of ascorbate peroxidase and removes excess H_2_O_2_ from plants. In this experiment, the ASA content decreased under the existence of Cd stress, which may be due to oxidative stress in plants under Cd stress [43]. The ASA consumption could lead to a decrease in the ASA content [44]. The increase in the ASA content by the inoculation of Cd-tolerant bacteria could be due to promoting the synthesis of ascorbic acid and the elimination of H_2_O_2_ in plants. Hence, the adverse impact of Cd stress on plants could be alleviated [45]. Glutathione can exert antioxidant effects through the ascorbate–glutathione cycle [46]. Zhang et al. [47] found that, with the increase in Cd concentration, the GSH content of ultra-black glutinous maize showed a decreasing trend, which was similar to our experiment. The decrease in glutathione content in this experiment could be attributed to the Cd-tolerant bacteria, which could not prevent the induced expression of Cd on the HO-1 gene, leading to the depletion of GSH [48]. It is also possible that Cd-tolerant bacteria alleviated the stress of Cd on ryegrass through other pathways. The response of glutathione to Cd weakened and the glutathione content decreased [49].

### 3.2. Effects of Cd-Tolerant Bacteria on Microbial Activity

Soil basal respiration is a major attribute related to soil fertility [50], which is commonly used to measure soil quality. It has been reported in the literature that soil basal respiration is decreased with the increase in Cd concentration [51]. This effect could originate from the fact that Cd has an adverse impact on soil microorganisms and reduces the metabolic activities of soil microorganisms. Soil respiration significantly decreased in the late stage of inoculation with *R. erythropolis* in Cd-contaminated soil, possibly because the *R. erythropolis* inoculants increased the bioavailability of toxic compounds. The latter was achieved by producing surface-active compounds that enhanced the toxicity of the contaminant environment and affected soil microorganisms [52]. In this experiment, the inoculated Cd-tolerant bacteria reduced the soil basal respiration under Cd stress. This effect stems from the competition of soil microorganisms induced by inoculation of Cd-tolerant bacteria and the decrease in the soil microbial abundance, as well as the weakening of the soil respiration intensity.

Soil enzyme activity can accurately reflect the response of soil microorganisms to heavy metal exposure during phytoremediation [53]. Soil urease could affect the content of soil organic matter and nitrogen conversion [54], which can catalyze the hydrolysis of urea into ammonia and provide nitrogen for soil microorganisms. According to the literature, urease activity in the soil increases for a certain range of Cd concentration compared to unpolluted conditions [55]. This effect is in direct line with our results, suggesting that plant-root exudates promote the improvement of soil enzyme activity, nutrient cycling, and the survival of microorganisms in soil [56]. He et al. [57] pointed out that the inoculation with EE2-degrading bacteria (*Hyphomicrobium* sp.) could increase the soil urease activity. The latter originates from the promotion of the growth of nitrogen-transforming bacteria in soil.

Sucrase is a hydrolase that catalyzes the hydrolysis of sucrose into glucose and fructose. It is widely distributed in plants, microorganisms, and soils. Its substrates and products are the most abundant soluble sugars in soil, which provide the necessary energy for microorganisms, animals, and plants. It also plays an important role in the conversion of soil carbon and nitrogen [58]. The activities of sucrase and dehydrogenase first increased and then decreased under the existence of the Cd stress. This may be due to the excitatory effect of toxicants, which takes place when the organism is stimulated by external pollutants. As a result, a compensation process in the organism could possibly exist. When the impact of pollutants on the organism is low, the compensation effect is higher than the impact of pollutants, which promotes the growth of the organism. With the increase in the pollutant concentration, the inhibitory effect of the pollutants exceeds the compensatory effect of organisms, and the growth of organisms gets inhibited [59]. Studies have shown that inoculated bacteria strains can enhance soil sucrase activity [60,61], which is consistent with the results of this experiment. The increased sucrase activity via inoculating Cd-tolerant bacteria could be due to the decomposition of certain substances in the soil by the tolerant bacteria, which provide nutrients for the microbial community [62].

The dehydrogenase activity is an important index to measure the toxicity of heavy metals in contaminated soil [63]. Dehydrogenase is an essential enzyme in living cells, involved in redox reactions that promote the dehydrogenation of soil organic matter and transfer energy to hydrogen receptors through the respiratory chain [64]. The addition of low Cd can stimulate soil microbial biomass and its metabolic activity [65]. In this experiment, the inoculation of Cd-tolerant bacteria reduced the dehydrogenase activity because the Cd-tolerant bacteria changed the soil environment, the pH, and the redox property of the soil, as well as inhibited the dehydrogenase activity. The inoculation of Cd-tolerant bacteria weakened the soil oxidative stress response and declined the enzyme activity accordingly.

### 3.3. Effects of Cd-Tolerant Bacteria on Soil Cd Forms and Cd Accumulation of Ryegrass

In this experiment, the inoculation of Cd-tolerant bacteria changed the Cd morphology in soil. The adverse effect of heavy metals on plants is not only related to the content of heavy metals but also to the form of heavy metals in soil. It is generally believed that the bioavailable Cd and the exchangeable Cd have high biological activity, which could be easily absorbed by animals and plants. Residual Cd has low bioavailability and weak migration ability and, hence, cannot be readily absorbed by animals and plants [66]. Liu et al. [67] proved that the combined treatment of arbuscular mycorrhiza and biochar amendments had a synergistic effect on inducing soil Cd immobilization, reducing plant availability of Cd, and imposing the risk of post-harvest transfer. Under the treatment of 4 mg/kg Cd, the contents of exchangeable and potential bioavailable forms of Cd in soil increased by inoculating Cd-tolerant bacteria. This effect was conducive to Cd enrichment in plant roots and the reduction of Cd in soil, which was identical to that of Sun et al. [68]. Under the application of 20 mg/kg Cd contamination, the proportions of exchangeable Cd and residual Cd in the soil inoculated with Cd-tolerant bacteria were lower than those in the soil without inoculating with Cd-tolerant bacteria. The potential bioavailability of Cd increased, which may be because the inoculation of Cd-tolerant bacteria promoted the transformation between different forms of Cd. It has been reported that the inoculation of metal-resistant PGRP on rice reduces the concentration of Cd in the soil [69]. This result indicates that the inoculation of Cd-tolerant bacteria is conducive to the absorption of Cd in soil by plants.

With the increase in the cadmium concentration in soil, the accumulation of cadmium in ryegrass increased, which reflected the high tolerance and enrichment ability of ryegrass to Cd [70]. Hu et al. [71] suggested that the Cd absorbed by ryegrass was mainly stored and distributed in the roots, which is in direct line with the results of this experiment. The inoculation of Cd-tolerant bacteria can generally improve the enrichment of Cd in ryegrass, which is consistent with the results obtained by Guo et al. [72], who had inoculated *Burkholderia* sp. D54 on ryegrass. Yung et al. [73] also revealed that the inoculation with Pr27 and Pr30 strains increased the amount of Cd absorbed by plants by 90%. The strain could increase the bioavailability of metal in the soil. This effect could also originate from the increased biomass of ryegrass and heavy metal accumulation, which is positively correlated with plant biomass [74]. Therefore, the Cd accumulation of ryegrass increased, which was also consistent with the decrease in the soil’s residual Cd content in this experiment.

## 4. Materials and Methods

### 4.1. Materials

The soil was collected from a field having a clean soil layer of 0–20 cm. Stones, plant residues, and large and medium-sized soil animals were removed after collecting fresh soil and then air-dried and screened through a 2 mm sieve. The soil pH was 5.51. The total nitrogen content was 0.81 g/kg. The total phosphorus content was 0.48 g/kg. The total potassium content was 1.63 g/kg. Moreover, Cd was not detected. When determining soil pH, 1 g of soil was extracted with 2.5 mL of distilled water and measured with a pH meter. The total N of soil was determined by using an elemental analyzer. The total K was extracted by NaOH and determined by performing flame photometry measurements [75]. The total P was extracted with hydrofluoric acid and perchloric acid, and its content was determined by using the colorimetric method [76].

The plant was ryegrass (*Lolium perenne* L.), and the ryegrass seeds were purchased from Jiangsu Suqian City Horticulture Company Limited (Jiangsu Suqian, China).

The bacterial strain was screened from the contaminated soil of Fenghuang Mountain (30°53′32″ N, 118°1′21″ E), Tongling City, Anhui Province. It was identified as *Cellulosimicrobium* sp. Meanwhile, the strain was deposited in the China General Microbiological Culture Collection Centre as strain number CGMCC No. 25219, and the sequence of the partial 16S rRNA gene of that strain was deposited in the GenBank database under accession number PP204244 (please visit https://www.ncbi.nlm.nih.gov/nuccore/PP204244), accessed on 30 January 2024.

### 4.2. Experimental Design

A total of 5 treatments (Table 2) were set up in this experiment, with 3 replicates per treatment and a total of 15 pots. The test soil was contaminated by adding Cd in the form of a CdCl_2_·2.5H_2_O salt solution, and 0.5 g/L Cd^2+^ were accurately prepared as the mother liquid and evenly poured into the potting soil. According to the soil environmental quality standard of China (GB15618-2018 [77]), the Cd concentration (measured by Cd^2+^) reached 0, 4 mg/kg (control value of farmland soil), and 20 mg/kg (screening value of Class I construction land) after aging for 60 days. Each pot (8 cm × a8 cm × 10 cm) was filled with 500 g (dry weight) of soil, and 50 ryegrass seeds were evenly planted in each pot. According to the conventional application amount of N (300 kg/hm^2^) and P (150 kg/hm^2^), the water-soluble fertilizer was diluted and applied as a base fertilizer. Each pot was inoculated with 9 mL of Cd-resistant bacteria (10^9^ CFU/mL), illuminated for 14 h/d, and darkened for 10 h/d. The indoor culture experiment was carried out for 40 days. According to the soil-moisture condition of each pot, distilled water was weighed and added every day to maintain the potting soil’s moisture content at 60% of the soil’s saturated water content. Sampling was performed on day 40, and then, the plant physiology and biochemistry, soil microbial activity, and heavy metal content were analyzed.

### 4.3. Methods

#### 4.3.1. Determination of Physiological and Biochemical Characteristics of Ryegrass

The biomass was measured by the drying method [78]. The samples were placed in an oven at 105 °C for 30 min, then baked at 80 °C to a constant weight and weighed to obtain the biomass.

The chlorophyll content was determined by using the ethanol-grinding method [79]. Initially, 0.2 g of the cut fresh sample were weighed, and a small amount of quartz sand and calcium carbonate powder and 2–3 mL 95% ethanol were added and ground until it homogenized. Then, it was filtered with 95% ethanol into a 25 mL brown volumetric bottle and, finally, filled with ethanol to 25 mL and shaken well. Next, the chlorophyll was extracted into a colorimetric cup with a light diameter of 1 cm. The absorbance was determined at 665 nm and 649 nm, with 95% ethanol as a blank.

Superoxide dismutase (SOD) was determined by nitrogen blue tetrazole colorimetry [80]. First, 0.5 g of plant leaves (remove coarse leaf veins) in a pre-cooled mortar were taken, and 1 mL of pre-cooled phosphoric acid buffer on an ice bath was added to grind into pulp. Subsequently, the mixture was centrifuged at 4000 r/min for 10 min, and the supernatant was the crude extract of SOD. In a 10 mL centrifuge tube, 1.5 mL 0.05 mol/L pH = 7.8 phosphate buffer, 0.3 mL 130 mmol/L methionine solution, and 0.3 mL 750 μmol/L NBT solution were injected into the sample group successively. The tube contained 0.3 mL 100 μmol/L EDTA-Na_2_, 0.3 mL 20 μmol/L riboflavin solution, 0.05 mL enzyme solution, and 0.25 mL ultra-pure water. The enzyme solution was replaced by slow liquid in the control group. After mixing, half of the control group’s measuring tubes were placed in the dark. The absorbance of the other tubes was measured at 560 nm after the reaction under 4000 lx sunlight for 20 min.

The peroxidase (POD) activity was determined by guaiacol colorimetry [81]. First, 0.2 g of leaves were taken, cut into pieces, and ground until homogenized with ultra-pure water, The volume was set to 25 mL and were shaken well. Next, they were centrifuged for 1000 r/min for 20 min, and the supernatant was the enzyme liquid to be measured. A clean glass tube was taken, and 1.0 mL 0.1% guaiacol, 1.0 mL 0.18% H_2_O_2_, 1.0 mL enzyme solution, and blank control plus 1.0 mL ultra-pure water were added. The mixture was shaken well to accurately react at 25 °C for 10 min. Finally, 0.2 mL of 5% metaphosphoric acid was added to terminate the reaction. Absorbance was measured at 470 nm.

Glutathione (GSH) was determined by the 2-nitrobenzoic acid method [82]. First, 1 g of leaves was taken and placed in a mortar. Next, 50 g/L TCA extract pre-cooled at 4 °C in batches were added and ground into homogenate in an ice bath. The mixture was centrifuged at 12,000 r/min for 20 min, and the supernatant was the liquid to be measured. In the test tube, 1 mL supernatant, 1 mL 0.1 mol/L pH = 7.7 phosphoric acid buffer, and 1 mL 4 mmol/L DTNB solution were successively added, and the absorbance was measured at 412 nm after the reaction at 25 °C for 10 min.

Ascorbic acid (ASA) was determined by the colorimetric method [83]. Initially, 1.0 g of leaves was placed in a mortar, and 50 g/L TCA extract pre-cooled at 4 °C in batches were added. The mixture was ground into a homogenate in an ice bath, and 50 g/L TCA at a constant volume of 10 mL were used. Next, it was well mixed and filtered, and the collected filtrate was used. In the test tube, 1 mL filtrate, 1 mL 50 g/L TCA, 1 mL anhydrous ethanol, 0.5 mL 0.4% phospho-ethanol solution, 1 mL 5 g/L Rhodophenanthroline–ethanol solution, and 0.5 mL 0.3 g/L FeCl_3_-ethanol solution were successively added. When the reaction at 30 °C was completed after 60 min, the absorbance was measured at 534 nm.

#### 4.3.2. Determination of Soil Microbial Activity

The soil basal respiration was measured by the alkali absorption method [84]. The ampoule containing a 10 mL 0.05 mol/L NaOH solution was placed in a glass jar, sealed tightly, cultured at 25 °C for 24 h, and then, removed. Then, the NaOH solution was washed into a 100 mL conical bottle with CO_2_-free water, and 2 mL barium chloride was added. And, 2 drops of phenolphthalein indicator were titrated with 0.05 mol/L until the purplish red color disappeared. The soil basal respiration was expressed as CO_2_ released per gram of soil after 24 h (mg/g/d).

The soil sucrase was determined by the 3,5-dinitrosalicylic acid color development method [85]. First, 2.50 g of fresh soil was accurately weighed into a triangular bottle. Next, 0.5 mL toluene, 15 mL 8% sucrose solution, and 5 mL phosphoric acid buffer with pH = 5.5 were added, mixed well, and cultured in a constant temperature incubator at 37 °C for 24 h. Then, the mixture was rapidly filtered and absorbed 1 mL of filtrate. Then, 3 mL DNS reagent were added. The water bath was boiled for 5 min and a water-cooling termination for 3 min with a distilled water fixed volume of 50 mL was performed. The absorbance was determined at 508 nm. No matrix control was set for each soil sample, and no soil control was performed for the whole experiment to eliminate the error caused by sucrose originally existing in the soil. The soil sucrase activity was expressed as the number of milligrams of glucose produced per gram of soil after 24 h (mg/g/d).

The soil dehydrogenase was determined by the 2,3,5-triphenyltetrazolium chloride reduction method [86]. First, 2.00 g of fresh soil were accurately weighed into the colorimetric tube. Next, 2 mL TTC solution, 2 mL glucose solution, and 2 mL Tris buffer were added, shaken well, and cultured in an incubator (37 °C) for 24 h. A small amount of methanol was extracted many times, filtered in the colorimetric tube, and methanol was used to reach 50 mL. The absorbance was measured at 485 nm.

The soil urease was determined by the phenol-blue colorimetric method [87]. Initially, 2.50 g of fresh soil were accurately weighed into a triangular bottle. Then, 0.5 mL toluene first were added and reacted for 10 min. Next, 10 mL 10% urea and 20 mL citrate buffer were added, mixed well, cultured in a 37 °C incubator for 24 h, and filtered, whereas 1 mL filtrate was added into a colorimetric tube. Four mL sodium phenol solution and 3 mL sodium hypochlorite solution were successively added, and after color development for 20 min, the absorbance was determined at 578 nm by shaking with distilled water to 50 mL. No matrix control was set for each soil sample, and the same volume of ultra-pure water was used to replace the matrix. The other operations were the same as the sample test. At the same time, no soil-sample control should be done for the whole test to eliminate the error caused by the original ammonia in the soil. Soil urease activity was expressed as mg of NH_3_-N produced per gram of soil after 24 h (mg/kg/d).

#### 4.3.3. Determination of Cd Content

The content of Cd in the soil samples was determined by the HCl-HNO_3_-HF-HClO_4_ system. First, the soil sample of 0.2500 g (over 100 mesh sieve) was weighed and dissolved in a Teflon crucible on a controlled electric heating plate in the fume hood (containing 0.5 mL water, 5 mL hydrochloric acid, and 5 mL nitric acid). After heating at 110 °C for 1 h, it was taken off and slightly cooled. Next, 4 mL hydrofluoric acid and 1.5 mL perchloric acid were added, and the mixture was heated at 200 °C for 1.5 h. Then, the cover of the crucible was removed, and the heating was continued for 1.5 h. After that, it was heated at the temperature value of 380 °C to drive the acid until the white smoke was gone. Fifty percent of the nitric acid solution was added to warm and dissolve the residue, and the constant volume was 50 mL. Double filter paper was used to filter, and the cadmium content was determined by atomic absorption spectrophotometer. The Cd from the soil was extracted by the Tesseier multi-stage continuous extraction method [88].

The Cd content of the plant was digested by HNO_3_-HClO_4_. The cadmium content was determined by the atomic absorption spectrophotometer [89]. Initially, 0.2 g of dried 60-mesh sieve plant samples were weighed and put into a 100 mL triangle bottle, and 15 mL of mixed acid (HNO_3_:HClO_4_ = 4:1) were added. The bottle was sealed with plastic wrap and allowed to rest overnight. The stepwise heating method was used to digest on an electric heating plate the next day. The plastic wrap was removed, and the funnel was covered and heated up to 120 °C. Then, it was heated up to 180 °C after 30 min and to 240 °C after 30 min. The solution was digested until it became clear and transparent and cooled to room temperature. Finally, a volume of distilled water of 50 mL was to be measured.

#### 4.3.4. Identification of Tolerant Bacteria

The bacterial genome DNA of the strain was extracted by using the high salt method [90]. More specifically, 16S rDNA cloning, sequencing, and comparison processes concerning strains were carried out. Using the extracted total bacterial DNA as a template, the 16S rDNA universal primer was extracted. The forward primer was 5′-AGA GTT TGA TCC TGG CTC AG-3′ (27F). The reverse primer 5′-TAC CTT GTT ACG ACT T-3′ (1492R) was used to perform a 50 μL PCR reaction. Next, the PCR product was recovered, and the enzyme was connected to the pMD19-T vector (Takala, Japan) and, then, cloned for sequencing [91]. The sequencing was completed by Shenggong BioEngineering (Shanghai, China) Company Limited. Homology analysis was performed between the sequencing results and the corresponding 16SrDNA sequences on GenBank and its RDP. The 16SrDNA sequence of the type strain with close homology to the tested strain was compared from the NCBI database and identified as *Cellulosimicrobium* sp.

### 4.4. Instruments

A tissue grinder, microplate reader, graphite digester, TAS-990 AFG atomic absorption spectrophotometer (Beijing Puxi General Instrument Company Limited, Beijing, China), element analyzer, pH meter, centrifuge, thermostat, oscillating mixer, and MG48+ PCR instrument, BG-Power 600i Electrophoresis Instrument were used.

### 4.5. Data Analysis

SPSS27 was used for conducting a one-way ANOVA analysis of variance, and significant differences were determined by the Duncan test (*p* < 0.05). The Excel2016 and Origin2021 software packages were used for data statistics and plotting. The correlation between Cd accumulation and inoculated microorganisms, physiology, biochemistry of ryegrass, and microbial activity was evaluated by a Pearson correlation analysis.

## 5. Conclusions

By performing a set of experiments, it was found that the inoculation of Cd-tolerant bacteria successfully alleviated the stress of Cd pollution on ryegrass. Therefore, the peroxidase activity and glutathione content in ryegrass leaves increased. The Cd-tolerant bacteria enhanced the chlorophyll content in ryegrass leaves, improved the photosynthesis ability of plants, increased the ascorbic acid content, and removed excess H_2_O_2_ in ryegrass. The Cd-tolerant bacteria can change soil microbial activity, increase soil urease and sucrase activities, decrease soil basic respiration and dehydrogenase activities, and change soil Cd morphology. The enrichment of Cd in both above-ground and under-ground parts of ryegrass could be also increased. Therefore, the inoculation of Cd-tolerant bacteria can remarkably improve the ability of ryegrass to repair Cd-contaminated soil.

## Figures and Tables

**Table 1:** Contents of various forms of cadmium in soil (mg/kg).

Treatment	Exchangeable	Potential Bioavailability	Residual
C1	2.501 b	0.295 b	0.186 ab
C1B	2.637 b	0.340 b	0.026 b
C2	13.522 a	2.868 a	0.571 a
C2B	12.038 a	3.627 a	0.319 ab

Different letters in the same column indicated significant differences in different treatments (*p* < 0.05).

**Table 2:** Experimental design.

Treatment	Cd Pollution Concentration (mg·kg^−1^)	Cd Tolerant Bacteria
CK	0	Without inoculation
C1	4	Without inoculation
C1B	4	Inoculation
C2	20	Without inoculation
C2B	20	Inoculation

## Data Availability

All data generated or analyzed in this study are included in the published article.
